# Use of cognitive enhancers for mild cognitive impairment: protocol for a systematic review and network meta-analysis

**DOI:** 10.1186/2046-4053-1-25

**Published:** 2012-05-30

**Authors:** Andrea C Tricco, Charlene Soobiah, Erin Lillie, Laure Perrier, Maggie H Chen, Brenda Hemmelgarn, Sumit R Majumdar, Sharon E Straus

**Affiliations:** 1 Li Ka Shing Knowledge Institute, St. Michael’s Hospital, 209 Victoria Street, East Building, Toronto, ON, M5B 1T8, Canada; 2 Division of Nephrology, Department of Medicine, University of Calgary, Calgary, Canada; 3 Division of General Internal Medicine, Department of Medicine, University of Alberta, Edmonton, Canada; 4 Department of Geriatric Medicine, University of Toronto, Toronto, Canada

## Abstract

**Background:**

Elderly individuals who have memory problems without significant limitations in activities of daily living are often diagnosed as having mild cognitive impairment (MCI). Some of these individuals progress to dementia. Several cognitive enhancers (for example donepezil, galantamine, rivastigmine, memantine) have been approved for use in people with Alzheimer’s dementia but their use in patients with MCI is unclear. We aimed to determine the comparative effectiveness, safety, and cost of cognitive enhancers for MCI through a systematic review and network (that is, indirect comparisons) meta-analysis.

**Design/Methods:**

We will include studies that examine the use of cognitive enhancers compared to placebo, supportive care, or other cognitive enhancers among patients diagnosed with MCI. Outcomes of interest include cognition and function (primary outcomes), as well as behavior, quality of life, safety, and cost (secondary outcomes). We will include all experimental studies (randomized controlled trials, quasi-randomized controlled trials, controlled clinical trials), quasi-experimental studies (controlled before-after, interrupted time series), and observational studies (cohort, case–control). Studies will be included regardless of publication status (that is, we will include unpublished studies), year, or language of dissemination.

To identify potentially relevant material, we will search the following electronic databases from inception onwards: MEDLINE, Cochrane Central Register of Controlled Trials, EMBASE, CINAHL, and Ageline. The electronic database search will be supplemented by scanning the reference lists of included studies, searching Google and organization websites for unpublished or difficult to locate material literature, and contacting experts.

Two reviewers will independently screen the studies for inclusion using the eligibility criteria established *a priori* and independently extract data. Risk of bias will be assessed using the Cochrane Risk of Bias tool for experimental and quasi-experimental studies and the Newcastle Ottawa Scale for observational epidemiology studies. Meta-analysis and network meta-analysis are planned, if the studies are deemed statistically, methodologically, and clinically homogenous.

**Discussion:**

Our systematic review will provide important information regarding the benefits, costs, and harms of cognitive enhancers for patients with MCI. This information can be used to assist healthcare providers, policy-makers, MCI patients and their family regarding the use of these agents.

**PROSPERO registry number:**

CRD42012002234

## Background

Mild cognitive impairment (MCI) is a diagnosis reserved for elderly individuals who have memory problems without significant activities of daily living limitations [1]. The diagnostic criteria for MCI include: subjective complaint of memory loss; memory impairment on brief cognitive or neuropsychological testing; decline from previously normal level of function; present basic daily functioning unchanged; no medical, neurologic or psychiatric explanation for memory loss; and, cognitive impairment not meeting the criteria for dementia [1]. MCI has recently been recognized as a distinct condition.

A recent systematic review estimated the prevalence and incidence of MCI reported in population-based studies [2]. Ten studies conducted in China, France, Italy, Korea, and the United States reported that the prevalence of MCI ranged from 3% to 42%. The prevalence of MCI increased with age. For individuals 65 years of age or older, the incidence of MCI ranged from 21.5 to 71.3 per 1,000 person-years based on data from two studies conducted in the United States and one study conducted in Italy [2]. These estimates suggest that a large proportion of the population is affected by MCI. Patients with MCI may progress to dementia, thus there is interest in initiating treatment that may prevent or slow this progression.

Pharmacological treatment for Alzheimer’s dementia (AD) includes cholinesterase inhibitors (for example donepezil, galantamine, rivastigmine), as well as an N-Methyl-D-aspartic acid (NMDA) receptor antagonist, memantine (Ebixa, Lundbeck) [3]. Acetylcholinesterase inhibitors donepezil (Aricept, Eisai/Pfizer), rivastigmine (Exelon, Novartis), and galantamine (Reminyl, Shire) act by increasing the concentration of acetylcholine at the neurotransmitter sites [4]. Galantamine also modulates activity at nicotinic receptors [4]. Memantine works on the glutamatergic system and modulates the neurotransmitter glutamate [4]. Although cognitive enhancers have not been widely approved for patients with MCI and are available by special authorization in some countries (for example, Canada), patients and their family members are requesting these agents, making this a challenge in the primary and specialty care settings [1].

Given their increasing use, our objectives are to examine the comparative effectiveness, safety, and cost of cognitive enhancers for MCI through a systematic review and network meta-analysis (that is, indirect comparison meta-analysis). Specifically, our research questions are:

1. What is the efficacy of cognitive enhancers (donepezil, rivastigmine, galantamine, memantine) *vs.* placebo or best supportive care for MCI?

2. What is the comparative effectiveness of cognitive enhancers with similar modes of action (donezepil and rivastigmine) *vs.* galantamine and memantine for MCI?

3. What is the comparative safety of cognitive enhancers for MCI?

4. What is the comparative cost associated with cognitive enhancers for MCI?

## Methods/Design

This systematic review protocol was developed using guidance from the PRISMA Statement [5]. It was peer-reviewed by experts in pharmacology, statistics, and systematic reviews. Our protocol is registered in the PROSPERO database (CRD42012002234) and is closely-related to another systematic review protocol that focuses on cognitive enhancers for AD (CRD42012001948) (Tricco, unpublished paper submitted to BMC SRs).

### Eligibility criteria

Studies examining patients diagnosed with MCI using established criteria (for example, Montréal Cognitive Assessment [6] and DemTect [7] will be included. Studies must examine cognitive enhancers approved for use for AD in Canada (donepezil, rivastigmine, galantamine, memantine) compared with other cognitive enhancers, memantine or placebo and/or supportive care. These agents are also approved for AD in many other countries, including Australia, United States, and the United Kingdom. Supportive care will include social support, functional assistance, caregiver support, information, education, community service, and other non-pharmacological strategies.

We will include experimental studies (for example, randomized controlled trials [RCTs], quasi-randomized trials, controlled clinical trials), quasi-experimental studies (such as interrupted time series, controlled before and after studies), and observational epidemiology studies (for example, cohort, case–control studies). Inclusion will not be limited by publication status (that is, we will include unpublished material), year, or language of dissemination. Potentially relevant articles not written in English will be translated.

### Information sources and literature search

The main literature search will be conducted in the following electronic databases: MEDLINE (OVID interface, 1948 onwards), EMBASE (OVID interface, 1947 onwards), the Cochrane Methodology Register (current issue), Cochrane Central Register of Controlled Trials (CENTRAL, current issue), CINAHL (EBSCO interface, 1980 onwards), and Ageline (EBSCO interface, 1961 onwards). The search strategies will be developed using medical subject headings (MeSH) and text words related to cognitive enhancers for MCI patients.

The electronic database search will be supplemented by searching for difficult to locate or unpublished material (that is, grey literature) using methods (Tricco, unpublished paper submitted to BMC SRs). Briefly, we will search public health and trial registry websites, websites of organizations that produce guidelines, conference proceeding abstracts, Google, key journals, and contact manufacturers to obtain their Scientific Information Packets for the medications. Literature saturation will be ensured by searching the authors’ personal files, scanning the reference lists of included studies and relevant reviews, and contacting researchers and healthcare providers who are active in this field.

The literature searches will be conducted by an experienced librarian (Perrier) and peer-reviewed by another librarian using Peer Review of Electronic Search Strategies (PRESS) [8]. The draft literature search can be found in the Appendix. For simplicity, the literature search was conducted in conjunction with another systematic review on a closely-related topic (Tricco, unpublished paper submitted to BMC SRs). The two reviews will be separated after full-text screening. The results from the literature search will be uploaded to our online SysRev Tool, which will be used for screening the results from the literature search [9].

### Study selection process

We will calibrate the inclusion and exclusion criteria by pilot-testing a random sample of 50 citations resulting from the search. A kappa statistic and the percent agreement will be used to calculate inter-rater agreement for study inclusion between reviewers [10]. If poor to moderate agreement is observed (that is, percent agreement less than 70% or a kappa statistic less than 0.6), the inclusion and exclusion criteria will be revised. All citations and full-text articles will be reviewed by two reviewers independently. Conflicts will be resolved by discussion or the involvement of a third reviewer.

### Data items and data collection process

Key stakeholders (for example, patients, healthcare providers, policy-makers) will be engaged to refine the key outcomes. Potential outcomes of interest include:

Cognition: Mini-mental state examination, Goal Attainment Scale, Severe Impairment Battery

Function: Bristol Activities of Daily Living Scale, Caregiver-rated Modified Crichton Scale, Disability Assessment for Dementia, the Interview for Deterioration in Daily living activities in Dementia, Nurses Observation Scale for Geriatric Patients Activities of Daily Living subscale, the Progressive Deterioration Scale

Behavior: Neuropsychiatric Inventory

Global Status: Clinician Interview-Based Impression of Change Incorporating Caregiver Information scale, Clinical Global Impression of Change

Clinical Outcomes: Mortality, Health-Related Quality of Life, Institutionalization, Falls, Balance; Harms (number of adverse events (for example, nausea, vomiting, diarrhea, dizziness, weight loss, hospitalizations, bradycardia), number of withdrawals, number of withdrawals due to adverse events, severity and timing of adverse events); Benefits to caregivers (for example, caregiver stress)

Costs and cost-effectiveness

The data will be abstracted and categorized as study characteristics (for example, study design, year of conduct, setting), patient characteristics (such as type and number of patients, mean age, MCI diagnosis criteria, MCI severity, baseline cognition, co-morbidities), primary outcome results (for example, cognition, function), and secondary outcome results (such as behavior, quality of life, costs, falls, balance, and harms).

We will calibrate the online data extraction form by pilot-testing a random sample of five included studies. Two reviewers will abstract all data independently and conflicts will be resolved by discussion or the involvement of a third reviewer.

We will abstract data based on clinically and methodologically relevant follow-up time periods. These include after 3, 6, 12, and 24 months of follow-up. We will also identify multiple publications reporting data from the same group (that is, companion reports) to ensure that data is not counted twice in meta-analysis. Study authors will be contacted for further information when the data are unclear.

### Methodological quality/risk of bias appraisal

We will use the Cochrane Risk of Bias Tool to appraise RCTs [11] and the Cochrane Effective Practice and Organization of Care Risk of Bias Tool for controlled clinical trials, interrupted time series, and controlled before-after studies [12]. The Newcastle-Ottawa Scale will be used for cohort studies and case–control studies [13]. Summary of findings tables will be compiled to show the relevance and level of evidence across all of the included studies using Grading of Recommendations Assessment Development and Evaluation (GRADE) [14]. The McHarm tool [15] will be used to specifically examine adverse drug reactions reported in the included studies.

### Synthesis

We will first describe our results narratively. We will subsequently conduct meta-analysis using a random-effects model [16] for each type of study design separately. Clinical, methodological, and statistical heterogeneity will be examined and meta-regression analysis will be conducted if significant statistical heterogeneity is identified (that is, I^2^ statistic > 60%) [17]. Missing data will be imputed using established methods [18] and we will examine the effect of the missing data on our results using an established method [19].

If the data allow, network (such as, indirect comparison) meta-analysis will be conducted to rank treatment efficacy among all the available treatments using the WinBUGS software (MRC Biostatistics Unit, Cambridge, England) [20]. Sensitivity analyses will be conducted to assess the effect of inclusion of trials with high rates of dropouts, missing data imputations, instruments used for the primary outcomes, average adherence between groups, inclusion of observational studies in the network meta-analysis, and use of different priors for variance parameters in the network meta-analysis [21].

## Discussion

According to a recent systematic review, the prevalence of MCI increases with age, with population-based studies reporting prevalence estimates as high as 42% [2]. The incidence of MCI among seniors ranged from 21.5 to 71.3 per 1,000 person-years [2]. These estimates suggest that our systematic review results have the potential to influence a large proportion of the population. Our results will enable healthcare providers, policy-makers, MCI patients and their family members make decisions regarding the use of these agents.

As highlighted previously (Tricco, unpublished paper submitted to BMC SRs), we will employ a multifaceted knowledge translation strategy to ensure our results reach potential endusers. For example, we will publish our results in an open access journal present our findings at relevant meetings (such as the American Geriatrics Society). We will also disseminate our results through newsletters of interested organizations (for example, Alzheimer’s Society), create an educational module for healthcare providers and use our results to inform the development of a patient decision aid.

## Appendix: literature search

Database: Ovid MEDLINE(R) <1948 to November Week 2 2011>, Ovid MEDLINE(R) In-Process & Other Non-Indexed Citations < November 22, 2011> Search Strategy:

1. alzheimer$.mp.

2. “benign senescent forgetfulness”.mp.

3. binswanger$.mp.

4.(chronic adj2 cerebrovascular).mp.

5.(cognit$ adj2 (impair$ or declin$ or deficit$ or degenerat$ or deteriorat$ or los$ or disorder$ or complain$ or disturb$)).mp.

6.(cerebr$ adj2 (impair$ or declin$ or deficit$ or degenerat$ or deteriorat$ or los$ or disorder$ or complain$ or disturb$)).mp.

7. (memory adj2 (impair$ or declin$ or deficit$ or degenerat$ or deteriorat$ or los$ or disorder$ or complain$ or disturb$)).mp.

8.(mental adj2 (impair$ or declin$ or deficit$ or degenerat$ or deteriorat$ or los$ or disorder$ or complain$ or disturb$)).mp.

9.(ne?rocognit$ adj2 (impair$ or declin$ or deficit$ or degenerat$ or deteriorat$ or los$ or disorder$ or complain$ or disturb$)).mp.

10.(ne?ro-cognit$ adj2 (impair$ or declin$ or deficit$ or degenerat$ or deteriorat$ or los$ or disorder$ or complain$ or disturb$)).mp.

11.((cognit$ or memory or cerebral or brain) adj2 (improv$ or enhanc$ or perform$ or process$ or function$ or rehabilitation or aid$ or stimulat$)).mp.

12. cognition.ti.

13.(confusion$ or confused).tw.

14. dement$.mp.

15. deliri$.mp.

16. “ischemic white matter”.mp.

17.(“normal pressure hydrocephalus” and shunt$).mp.

18. “organic brain disease$”.mp.

19. “organic brain syndrome”.mp.

20. presenil$.tw.

21. pre-senil$.tw.

22. senil$.tw.

23. Alzheimer Disease/

24. Cognition Disorders/

25. Cognition/de [Drug Effects]

26. Confusion/

27. Delirium, Dementia, Amnestic, Cognitive Disorders/

28. Delirium/

29. Dementia/

30. Memory Disorders/

31. (aMCI or MCI).mp.

32. (“AA CD” or AACD).tw.

33. (“AA MI” or AAMI).tw.

34. ARCD.tw.

35. (“CI ND” or CIND).tw.

36. LCD.tw.

37. (MCD or MNCD).tw.

38. “M-MCI”.tw.

39. “N-MCI”.tw.

40. or/1-39

41. abixa.tw.

42. aricept.tw.

43. (acetylcholinesteraseadj inhibitor$).mp.

44. axura.tw.

45. akatinol.tw.

46. anti-cholinesterase?.tw.

47. anticholinesterase?.tw.

48. (cognitiveadjenhanc$).mp.

49. (cholinesteraseadj inhibitor$).mp.

50. ChEI.tw.

51. donepezil.mp.

52. ebixa.tw.

53. eranz.tw.

54. exelon.tw.

55. galant?amin$.tw.

56. lycoremine.tw.

57. memantin$.tw.

58. memox.tw.

59. namenda.tw.

60. nimvastid.tw.

61. nivalin$.tw.

62. “N-Methyl-D-aspartic acid receptor antagonist$”.tw.

63. prometax.tw.

64. razadyne.tw.

65. reminyl.tw.

66. rivastigmine.mp.

67. exp Cholinesterase Inhibitors/

68. Galantamine/

69. Memantine/

70. 357-70-0.rn. [CAS Registry Numbers]

71.19982-08-2.rn.

72. 120011-70-3.rn.

73. 120014-06-4.rn.

74. 123441-03-2.rn.

75. or/41-74

76. 40 and 75

77. Animals/not (Animals/and Humans/)

78. 76 not 77

## Competing interests

The authors report no conflicts of interests.

## Authors’ contributions

ACT conceived the study, designed the study, helped obtain funding for the study, and helped write the draft protocol. SV helped write the draft protocol. LP developed the search strategies and edited the draft protocol. CS registered the protocol and edited the draft protocol. EL edited the draft protocol. MHC provided input into the design and draft of the protocol. BH and SM provided feedback on the protocol during its development. SES conceived the study, designed the study, obtained the funding, and helped write the draft protocol. All authors read and approved the final protocol.
